# Editorial

**Published:** 1872-06-01

**Authors:** 


MEDICAL EXAMINER.

*A Semi-Monthly Journal of Medical Sciences.*

EDITED BY

N. S. DAVIS, M. D., AND F. H. DAVIS, M. D.

Chicago, June 1st, 1872.

### EDITORIAL.

In our sketch of the doings of the recent meeting of the Illinois State Medical Society, we have aimed to give only such items of practical interest and value as were developed in the papers and discussions, without regard to the general miscellaneous business of the session. The length of the report crowds out all our intended editorial matter for this number. The following officers were elected for the ensuing year:

*President*, Dr. D. W. Young, of Aurora.

*First Vice-President*, T. D. Washburn, of Hillsboro.

*Second Vice-President*, F. C. Hotz, of Chicago.

*Assistant Secretary*, Dr. R. D. Bradley, of Bloomington.

The next annual meeting is to be held in Bloomington on the third Tuesday in May, 1873.

